# Exploring Multidisciplinary Approaches to Comorbid Psychiatric and Medical Disorders: A Scoping Review

**DOI:** 10.3390/life15020251

**Published:** 2025-02-06

**Authors:** Ștefăniță-Tiberiu Țenea-Cojan, Venera-Cristina Dinescu, Veronica Gheorman, Ioana-Gabriela Dragne, Victor Gheorman, Mircea-Cătălin Forțofoiu, Maria Fortofoiu, Adrian Gabriel Dobrinescu

**Affiliations:** 1 Department of Surgery, University of Medicine and Pharmacy of Craiova, 200349 Craiova, Romania; tiberiu.tenea@umfcv.ro; 2 Department of Health Promotion and Occupational Medicine, University of Medicine and Pharmacy of Craiova, 200349 Craiova, Romania; 3 Department of Medical Semiology, University of Medicine and Pharmacy of Craiova, 200349 Craiova, Romania; catalin.fortofoiu@umfcv.ro; 4 University of Medicine and Pharmacy of Craiova, 200349 Craiova, Romania; idragne@yahoo.com; 5 Department of Psychiatry I, University of Medicine and Pharmacy of Craiova, 200349 Craiova, Romania; victor.gheorman@umfcv.ro; 6 Department of Emergency Medicine, University of Medicine and Pharmacy of Craiova, 200349 Craiova, Romania; maria.fortofoiu@umfcv.ro; 7 Department of Thoracic Surgery, University of Medicine and Pharmacy of Craiova, 200349 Craiova, Romania; adrian.dobrinescu@umfcv.ro

**Keywords:** psychiatric disorders, internal diseases, mental health, integrated care, comorbidities, neuroinflammation, diagnostic challenges, holistic care, multidisciplinary care, psychosocial factors

## Abstract

Psychiatric disorders often coexist with internal medical conditions, posing significant challenges to diagnosis, treatment, and overall patient outcomes. This study examines the bidirectional relationship between these conditions, emphasizing their impact on morbidity, treatment adherence, and quality of life. Through a comprehensive review of the peer-reviewed literature, we explore the physiological, neuroinflammatory, and psychosocial mechanisms that underpin these interactions. Key psychiatric disorders, including depression, anxiety, cognitive impairments, and psychosis, are identified as critical contributors to diagnostic complexity and therapeutic hurdles. Our findings underscore the importance of integrated, multidisciplinary care models, advocating for early detection, routine mental health screening, and personalized treatment strategies. Challenges such as overlapping symptoms, diagnostic ambiguities, and potential drug interactions are critically analyzed, with practical, evidence-based recommendations proposed for clinical practice. Despite advancements, significant research gaps persist, particularly the lack of longitudinal studies and the limited application of precision medicine tailored to this population. Future directions focus on enhancing diagnostic tools, developing innovative therapeutic approaches, and integrating mental health services into routine care. This study highlights the critical need for holistic, patient-centered approaches to manage comorbid psychiatric and internal medical conditions, offering actionable insights to improve outcomes and bridge existing gaps in research and practice.

## 1. Introduction

**Figure 1:**
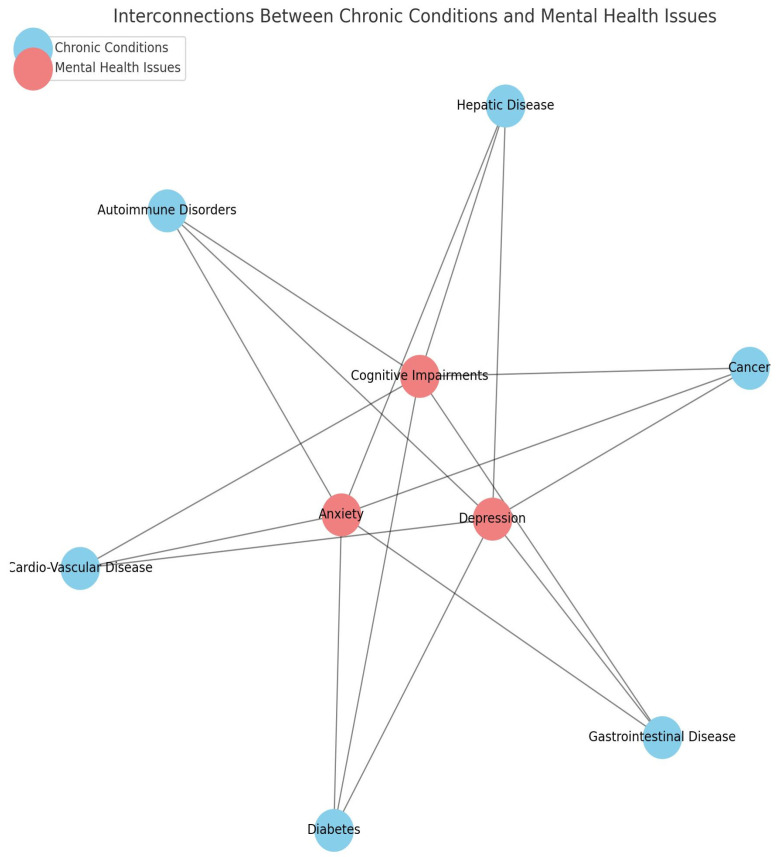
Interconnections between chronic conditions and mental health.

The prevalence and impact of psychiatric conditions in patients with internal dis-eases represent a complex and significant aspect of healthcare. Understanding the inter-play between physical and mental health is essential for providing comprehensive and effective patient care (Figure 1) [1,2,3,4,5].

Studies have consistently shown that psychiatric conditions are highly prevalent in patients with internal diseases [6,7]. For instance, individuals with chronic conditions such as hepatic disease, gastrointestinal disease, diabetes, cardiovascular disease, cancer, and autoimmune disorders often experience higher rates of depression, anxiety, and cognitive impairments compared to the general population [5,6,7]. Despite this, current clinical practices often overlook or inadequately address the integration of mental health assessments into the management of internal diseases. This creates a significant gap in holistic care approaches.

Research indicates that the prevalence of psychiatric conditions varies depending on the specific internal disease. For instance, a study conducted in Ethiopia found that 64.5% of cancer patients experienced psychological distress [8]. Similarly, individuals with chronic pain are at a heightened risk for mental health issues; approximately 35% to 45% of people with chronic pain experience depression [9].

The impact of psychiatric conditions on patients with internal diseases is profound, affecting both their quality of life and overall health outcomes. Mental health issues can exacerbate physical symptoms, reduce treatment adherence, and diminish the patient’s ability to cope with the challenges of their underlying medical condition. For example, patients with chronic pain have a threefold increased risk of developing psychiatric symptoms, such as mood or anxiety disorders [10,11,12].

Moreover, psychiatric conditions in patients with internal diseases are associated with poorer prognoses, longer hospital stays, increased healthcare utilization, and higher mortality rates. These patients often experience greater functional impairment and lower rates of treatment success. For instance, individuals with chronic pain are at a higher risk for suicide and suicidal thoughts, with research indicating that approximately 20% of people with suicidal ideation and between 5% and 14% of those who commit suicide suffer from chronic pain [13,14].

Research indicates that the prevalence of psychiatric conditions varies depending on the specific internal disease [9,10,11,12,13,14,15,16,17]. For example, patients undergoing treatment for cancer may experience heightened levels of distress, while those with chronic pain conditions may be at an increased risk for developing mood disorders [12,13,14].

**Figure 2:**
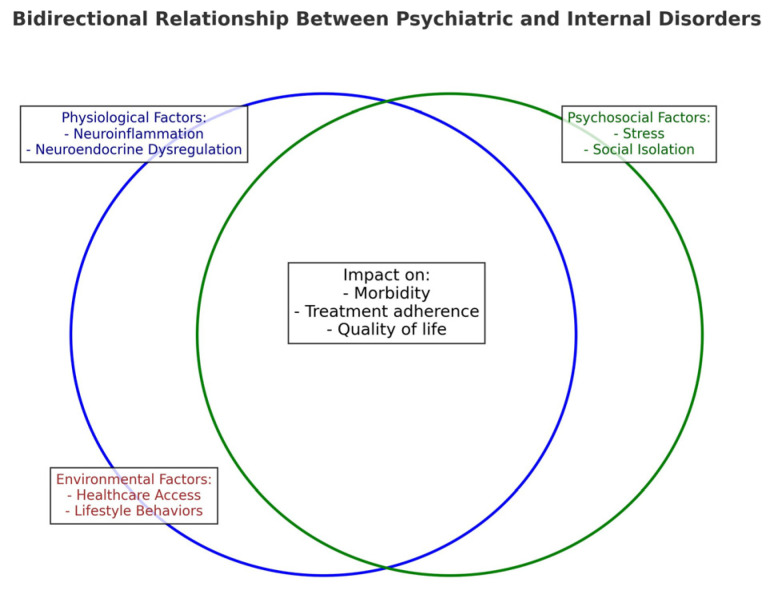
Bidirectional relationship between psychiatric and internal disorders.

The impact of psychiatric conditions on patients with internal diseases is profound, affecting both their quality of life and overall health outcomes (Figure 2). Mental health issues can exacerbate physical symptoms, reduce treatment adherence and diminish the patient’s ability to cope with the challenges of their underlying medical condition [10,11,12,13,14].

Psychiatric conditions in patients with internal diseases are associated with poorer prognoses, longer hospital stays, increased healthcare utilization, and higher mortality rates. Additionally, these patients often experience greater functional impairment and lower rates of treatment success [7,8,9,10,11,12,13,14,15,16].

Furthermore, the presence of psychiatric conditions can complicate the management of internal diseases by influencing medication adherence, treatment responses, and overall patient well-being [1,12,13,14,15,16,17,18,19]. Understanding the prevalence and impact of psychiatric conditions in this population is crucial for implementing integrated care approaches that address both physical and mental health needs. This recognition can lead to improved patient outcomes and a better overall quality of life for individuals managing internal diseases alongside psychiatric conditions

While there is substantial research on the prevalence and impact of psychiatric conditions in these populations, limited attention has been paid to the underlying bidirectional mechanisms and practical strategies for integrated management [1,2,14,15,16,17,18,19]. Specifically, the interplay of neuroinflammation, neuroendocrine dysregulation, and psychosocial stressors in shaping these comorbidities remains underexplored. Moreover, guidance on addressing diagnostic challenges, overlapping symptoms, and medication interactions is insufficient in the existing literature.

This review aims to fill these critical gaps by synthesizing current evidence on the relationship between psychiatric conditions and internal diseases, focusing on their bidirectional mechanisms and clinical implications. By emphasizing the need for multidisciplinary, patient-centered care, this review seeks to advocate for practical strategies that address the intertwined nature of mental and physical health. Our goal is to provide actionable insights to advance diagnostic and therapeutic practices, ultimately improving outcomes for patients with comorbid psychiatric and internal medical conditions.

## 2. Interconnections Between Psychiatric Conditions and Internal Diseases

### 2.1. The Relationship Between Psychiatric Conditions and Internal Diseases

**Figure 3:**
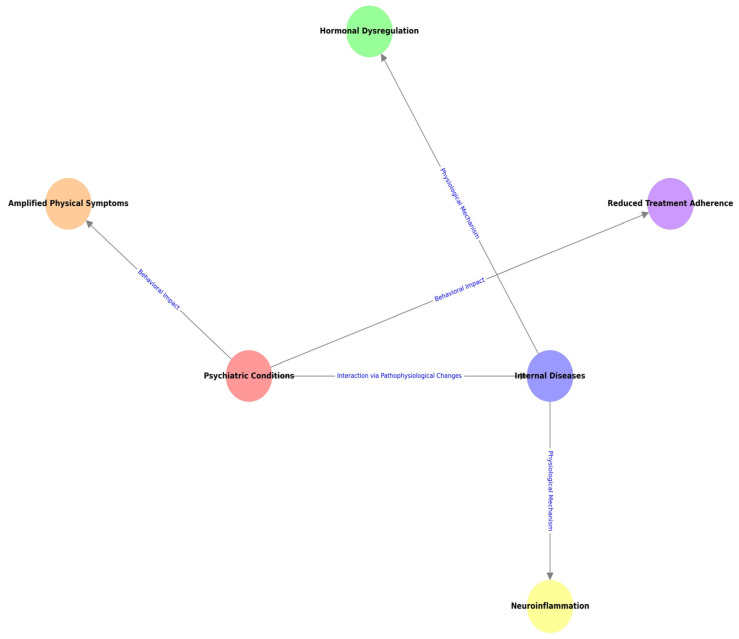
Interconnections between psychiatric conditions and internal disease.

The relationship between psychiatric conditions and internal diseases is multifaceted and bidirectional, shaping the onset, progression, and management of both categories of conditions (Figure 3). This intricate connection highlights how internal diseases can predispose individuals to psychiatric symptoms through physiological changes, such as neuroinflammation or hormonal dysregulation, while psychiatric conditions can exacerbate the course of internal diseases by reducing treatment adherence or amplifying physical symptoms. Recognizing and addressing this interdependence is fundamental for providing comprehensive, patient-centered care [3,4].

A wide spectrum of psychiatric symptoms is commonly observed in patients with various internal diseases, each with distinct manifestations and implications for clinical practice. Depression is one of the most prevalent psychiatric conditions associated with internal diseases [5]. Research consistently demonstrates its bidirectional relationship with conditions such as cardiovascular disorders, diabetes, and chronic pain. These comorbidities not only impair physical health outcomes but also increase the overall burden on healthcare systems.

Anxiety disorders similarly play a significant role in patients with chronic illnesses, including respiratory diseases, gastrointestinal disorders, and autoimmune conditions [6]. These disorders intensify the perception of physical symptoms, impede functional capacity, and complicate disease management. Moreover, psychosis, characterized by symptoms such as hallucinations and delusions, can arise in hepatic disorders, neurological conditions, or endocrine abnormalities [20]. The presence of psychotic symptoms demands a thorough understanding of both the underlying etiology and the interconnected psychiatric and medical challenges.

Mood disorders, particularly bipolar disorder, further illustrate the complexity of this relationship [2,3,4,5,6,16,20,21,22,23]. Bipolar disorder is frequently associated with metabolic syn-drome, cardiovascular diseases, and thyroid dysfunction, creating a bidirectional impact on both psychiatric and medical outcomes [16,21,22,23]. The oscillation between depressive and manic episodes in bipolar disorder can complicate disease management, as depressive episodes impair treatment adherence, while manic episodes increase the risk of engaging in harmful behaviors or neglecting medical care [22,23,24]. Integrated management strategies that address both psychiatric and medical aspects are crucial for improving outcomes in patients with these comorbid conditions.

Cognitive impairments, commonly observed in neurodegenerative and cerebrovascular conditions, also underscore the profound interplay between mental health and physical health [25]. These impairments necessitate personalized care plans that can adapt to the unique needs of each patient.

Furthermore, the mechanisms underlying this interplay are diverse and include physiological factors such as neuroinflammation, psychological stressors stemming from chronic disease, and environmental contributors like social isolation or limited access to healthcare. Understanding these mechanisms allows for the development of more effective diagnostic and therapeutic strategies, paving the way for integrated care models that optimize patient outcomes.

Overall, the intricate and reciprocal relationship between psychiatric conditions and internal diseases highlights the need for a holistic, interdisciplinary approach to care. By addressing both the physical and mental health needs of patients, clinicians can improve treatment outcomes, enhance the quality of life, and reduce the burden of comorbidity on individuals and healthcare systems alike.

### 2.2. Physiological Factors

**Figure 4:**
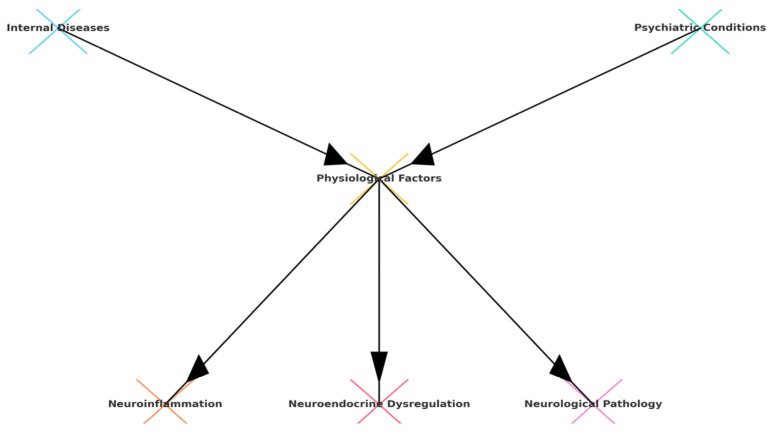
Key physiological mechanisms.

Understanding the physiological mechanisms underlying the interplay between internal diseases and psychiatric conditions is essential for elucidating the pathophysiology of comorbid symptoms. Key factors include neuroinflammation, neuroendocrine dysregulation, and neurological pathology (Figure 4).

Neuroinflammation: Internal diseases such as autoimmune disorders and chronic inflammatory conditions can provoke neuroinflammatory responses within the central nervous system. Proinflammatory cytokines and immune dysregulation have been implicated in alterations to brain function and mood regulation, contributing to psychiatric symptoms commonly observed in these patients [23].

Neuroendocrine dysregulation: Disruptions in the hypothalamic–pituitary–adrenal (HPA) axis and stress response systems are frequently observed in the context of internal diseases. Aberrant cortisol levels and impaired cortisol metabolism play a pivotal role in the pathogenesis of mood disorders and anxiety, linking internal diseases to psychiatric conditions [3].

Neurological pathology: Internal diseases such as neurodegenerative disorders and cerebrovascular conditions can directly affect brain structure and function. Structural brain changes and neuronal dysfunction are significant contributors to the development of cognitive impairments, mood disturbances, and psychosis in these patients [4].

### 2.3. Psychosocial Factors

Psychosocial factors play a critical role in shaping the mental health outcomes of patients with internal diseases [5]. Chronic illness not only imposes physical burdens but also triggers significant psychological and social challenges that can exacerbate psychiatric symptoms. Key psychosocial contributors include stress, illness perception, and the burden of disease-associated symptoms.

Psychosocial stress: Living with chronic or life-threatening internal diseases often precipitates psychological distress, leading to conditions such as depression and anxiety. The fear of mortality, disruption of personal identity, and experiences of social isolation significantly impact mental well-being, highlighting the profound psychosocial toll of chronic illness [6].

Illness perception and coping strategies: An individual’s perception of their illness and their ability to adapt to the associated physical limitations are pivotal for psychological well-being. Maladaptive coping mechanisms, such as avoidance or catastrophizing, along with feelings of helplessness, increase vulnerability to psychiatric conditions, including depression and anxiety [26].

Disease-associated symptom burden: Persistent physical symptoms such as pain, fatigue, and dyspnea directly influence mood and mental health. These symptoms not only contribute to the development of depressive and anxiety disorders but also perpetuate a cycle of psychological and physical distress, further compromising overall well-being [21].

### 2.4. Environmental Factors

Environmental factors play a pivotal role in influencing the mental health of patients with internal diseases [16]. These external influences, including social dynamics, healthcare accessibility, and lifestyle behaviors, contribute significantly to the psychological well-being of individuals, shaping their ability to cope with chronic illness.

Social support and stigma: The availability of robust social support networks can serve as a protective factor for mental health, while social isolation and stigma associated with specific internal diseases often exacerbate psychological distress. A perceived lack of understanding or support from others can contribute to the development of psychiatric conditions such as depression and anxiety, underscoring the importance of fostering inclusive and supportive environments [27].

Access to care and treatment adherence: The availability of healthcare resources, including mental health services, and the capacity to adhere to prescribed treatment regimens profoundly impact the mental well-being of patients. Barriers such as limited access to comprehensive care or mental health interventions can lead to poorer mental health outcomes, further complicating disease management [18].

Lifestyle and health behaviors: Lifestyle factors, including physical activity, dietary habits, and substance use, significantly affect both physical and mental health in patients with internal diseases. Unhealthy behaviors and maladaptive coping mechanisms can intensify psychiatric symptoms, hinder treatment adherence, and impede overall well-being [19].

The intricate interplay of environmental, physiological, and psychological factors underscores the complexity of the relationship between internal diseases and psychiatric conditions. Recognizing these interconnected mechanisms is essential for developing targeted, patient-centered interventions that address the holistic needs of individuals living with chronic illness [28].

### 2.5. Clinical Manifestations and Challenges 

The clinical manifestations of psychiatric conditions in patients with internal dis-eases pose unique diagnostic and therapeutic challenges [29,30,31]. These complexities arise from the overlap between psychiatric and physical symptoms, the bidirectional nature of their interactions, and the increased vulnerability of certain populations. Understanding these nuances is essential for delivering tailored and comprehensive care to this patient group.

Atypical symptoms: Psychiatric conditions in patients with internal diseases often present with atypical or nonspecific symptoms, which can overlap with physical manifestations of the underlying medical condition. For instance, depression or anxiety symptoms may be misinterpreted as part of the disease process or as a reaction to physical discomfort, leading to the under recognition and delayed diagnosis of psychiatric conditions [32].

Masked symptoms: Psychiatric distress in this population frequently manifests as “masked” symptoms, including unexplained somatic complaints, exacerbation of physical symptoms, or functional impairments. These presentations can obscure underlying psychiatric pathology, complicating the diagnostic process and delaying appropriate mental health interventions [33].

Bidirectional impact: The reciprocal relationship between psychiatric conditions and internal diseases exacerbates both physical and mental health symptoms. Psychiatric conditions, such as depression and anxiety, can negatively impact treatment adherence and disease management, resulting in poorer medical outcomes. Conversely, the burden of chronic physical illness can contribute to the onset or worsening of psychiatric symptoms, creating a cycle of mutual reinforcement [34].

Increased vulnerability: Certain populations, such as patients with neurodegenerative conditions or diseases affecting the central nervous system, are particularly vulnerable to developing psychiatric symptoms. This vulnerability arises from the direct impact of these diseases on brain function, necessitating specialized approaches to evaluation and management [35].

Addressing these challenges requires a holistic and interdisciplinary approach that accounts for the intertwined nature of psychiatric and physical symptoms. Early recognition, accurate diagnosis and integrated treatment strategies are critical for improving outcomes and enhancing the overall well-being of patients with internal diseases.

### 2.6. Differences Compared to Individuals Without Internal Diseases: [36]

Patients with internal diseases and psychiatric conditions face unique challenges that distinguish them from individuals without underlying medical conditions [36]. These differences stem from the intricate interplay between physical and mental health, requiring nuanced approaches to diagnosis, treatment, and prognosis.

Diagnostic challenges: The overlap between somatic and psychiatric symptoms com-plicates the accurate diagnosis of psychiatric conditions in patients with internal diseases. Atypical and masked presentations of psychiatric disorders are common, making it essential to differentiate between primary psychiatric conditions and secondary manifestations arising from the internal disease. This process requires careful evaluation and a thorough understanding of the patient’s medical history [37].

Treatment considerations: The coexistence of internal diseases and psychiatric conditions often necessitates tailored treatment approaches. This includes adjustments in medication selection and dosing to minimize the risk of drug interactions and side effects. Additionally, effective management of physical symptoms and functional limitations associated with internal diseases must be integrated into psychiatric care to optimize overall well-being [18].

Impact on prognosis: Psychiatric comorbidities significantly affect the prognosis and trajectory of internal diseases. These conditions are associated with increased healthcare utilization, higher complication rates, and poorer outcomes. Addressing mental health needs within the context of comprehensive disease management is crucial for improving overall patient outcomes [19].

Multidisciplinary collaboration: Patients with internal diseases and comorbid psychiatric conditions benefit from coordinated, multidisciplinary care. Managing the interconnected aspects of physical and mental health requires seamless teamwork between medical practitioners, mental health specialists, and allied healthcare providers. This approach ensures holistic, patient-centered care that enhances quality of life [1].

The interplay between psychiatric conditions and internal diseases underscores the need for nuanced and comprehensive approaches to assessment, diagnosis, and management. Addressing the challenges posed by this intersection is critical for delivering person-centered care that meets the interconnected needs of this population. Effective management requires not only the recognition of these unique complexities but also the integration of multidisciplinary efforts to achieve better patient outcomes [29].

#### Highlighting Challenges and Complexities in Diagnosis and Management

Diagnosing and managing psychiatric conditions in patients with internal diseases involves navigating the intricate interplay between physical and mental health. Unique difficulties arise from disease-specific symptoms, medication interactions, and the influence of the underlying medical condition on the patient’s mental status. These complexities call for targeted strategies that consider the full spectrum of a patient’s health needs [3,4].

### 2.7. Challenges in Diagnosis: [32]

Diagnosing psychiatric conditions in patients with internal diseases presents unique challenges due to the interplay between physical and mental health [32]. The overlapping nature of symptoms, atypical presentations, and diagnostic uncertainty often complicate the identification of psychiatric comorbidities in this population, requiring careful evaluation and specialized expertise.

Overlapping symptoms: Symptoms of internal diseases often mimic or mask psychiatric conditions, complicating the differentiation between primary psychiatric disorders and secondary manifestations related to the medical condition. For example, fatigue, sleep disturbances, and cognitive impairments are frequently attributed to the underlying disease, potentially obscuring concurrent psychiatric symptoms [38].

Atypical presentations: Psychiatric conditions in patients with internal diseases often deviate from classic symptom profiles, leading to delayed recognition and diagnosis. De-pression, for instance, may manifest predominantly as irritability or somatic complaints rather than the hallmark features of mood disturbance in patients with chronic pain [39].

Diagnostic uncertainty: The coexistence of complex medical disorders often results in diagnostic ambiguity, particularly in distinguishing between delirium, dementia, and primary psychiatric conditions. Cognitive impairments related to the underlying disease further complicate this process, necessitating the use of comprehensive assessment tools and input from specialists with expertise in neuropsychiatry [15].

### 2.8. Complexities in Management: [40]

Managing psychiatric conditions in patients with internal diseases presents unique challenges due to the interplay between physical and mental health [40]. Effective management requires careful consideration of treatment interactions, disease-specific symptoms, and barriers to adherence, all of which can complicate care and influence outcomes.

Treatment interactions: The coexistence of internal diseases and psychiatric conditions necessitates careful attention to potential drug–drug interactions and altered pharmacokinetics. Treatments for the primary medical condition may affect the metabolism or efficacy of psychotropic medications, while the underlying disease itself can lead to metabolic changes that impact medication safety and effectiveness [41]. For example, patients with end-stage kidney failure and bipolar disorder face significant pharmacological challenges. Renal impairment limits the use of many psychotropic medications, such as lithium, which requires renal clearance, increasing the risk of toxicity. Alternative treatment strategies, including the use of antipsychotics with minimal renal excretion, highlight the importance of personalized care and close collaboration between psychiatry and nephrology [42].

A significant barrier to the effective treatment of psychiatric comorbidities in patients with internal diseases is the limited awareness and knowledge among healthcare providers regarding psychotropic medications. Addressing this issue requires a multifaceted approach. Firstly, structured training programs should be implemented to enhance healthcare workers’ understanding of psychotropic drug indications, mechanisms of action, potential side effects, and drug–drug interactions. Collaborative care models, wherein psychiatrists and pharmacists support primary and tertiary care teams, can further improve confidence and competence in prescribing and managing these treatments. Additionally, the integration of decision-support tools within electronic health records can provide real-time alerts about potential medication interactions and guide optimal prescription practices. Continuous professional development initiatives, such as case-based learning and workshops, tailored to address specific challenges in managing psychiatric symptoms, are essential. By equipping healthcare providers with the necessary knowledge and tools, these strategies aim to bridge gaps in care and improve outcomes for patients with complex medical and psychiatric needs.

Disease-specific symptoms: Certain internal diseases, such as encephalopathies, endocrine disorders, and neurodegenerative conditions, may manifest with neuropsychiatric symptoms that complicate the assessment and management of psychiatric conditions. These cases require specialized evaluation and tailored interventions distinct from those used for primary psychiatric disorders [18].

Impact on treatment adherence: Patients with comorbid psychiatric conditions and internal diseases often face barriers to treatment adherence. Cognitive impairments, physical limitations, and the psychological burden of chronic illness can hinder adherence to both medical and psychiatric treatment plans. Addressing these barriers is critical for optimizing outcomes and ensuring effective disease management.

To address the gap in mental health screening within noncommunicable disease clinics, short tools such as the PHQ-2, PHQ-4, PHQ-9, and GAD-7 have been identified as effective and time-efficient instruments [43]. These tools are particularly suitable for high-burden clinical environments due to their brevity and validated ability to detect anxiety and depression. However, the implementation of these screening methods faces challenges, including the workload of healthcare providers, which may limit their capacity to administer initial assessments. To overcome this, task-sharing models, where trained auxiliary staff or nurses perform the screenings, could be considered. Additionally, integrating digital solutions, such as electronic tablets or online platforms, can streamline the process, allowing patients to complete the forms independently while waiting for consultations. Evidence from existing studies highlights that early detection of psychiatric symptoms significantly improves treatment adherence and clinical outcomes in patients with chronic diseases. Therefore, implementing such tools not only bridges a critical gap but also aligns with holistic, patient-centered care models. Future research should explore the feasibility of these strategies in diverse clinical settings, particularly in resource-constrained environments.

Successfully navigating these complexities requires an integrated, multidisciplinary approach that accounts for the interplay of physical and mental health factors. Tailored interventions and close monitoring are essential for improving patient outcomes and quality of life.

### 2.9. Influence of the Underlying Medical Condition

The underlying medical condition plays a pivotal role in shaping the presentation, progression, and management of psychiatric symptoms. Its influence extends across neurobiological, psychosocial, and treatment-related domains, underscoring the need for integrated care approaches [44].

Neurobiological effects: Many medical conditions exert direct neurobiological effects that contribute to or exacerbate psychiatric symptoms [45]. Neuroinflammatory processes, neurotransmitter imbalances, and structural brain changes associated with diseases such as autoimmune disorders or neurodegenerative conditions can significantly impact the development and expression of psychiatric comorbidities [45].

Disease burden: Living with a chronic or debilitating medical condition imposes a substantial psychosocial and emotional burden on patients. Functional limitations, persistent pain, social isolation, and the fear of disease progression can lead to the emergence of anxiety, depression, and other psychiatric symptoms [46]. These challenges highlight the importance of addressing the broader psychosocial context of the patient’s illness.

Treatment implications: The coexistence of a medical condition and psychiatric symptoms necessitates tailored management strategies. These may include adjustments to psychotropic medication regimens to account for potential drug–disease interactions, the use of psychotherapy approaches adapted to the patient’s physical limitations, and the provision of support services designed to address the unique challenges posed by the interplay of physical and mental health concerns [47].

A thorough understanding of the influence of underlying medical conditions is essential for providing holistic, patient-centered care. By addressing the neurobiological, psychosocial, and treatment-related complexities, clinicians can enhance the overall well-being and quality of life for patients with comorbid physical and psychiatric conditions.

### 2.10. Addressing the Challenges and Complexities

Effectively managing psychiatric conditions in patients with internal diseases re-quires an integrated approach that considers the interplay between physical and mental health. Key strategies include comprehensive assessment, individualized interventions, and collaborative care.

Comprehensive assessment: An integrative assessment that thoroughly evaluates both physical and mental health is fundamental. This process should involve a multidisciplinary team with expertise in medical and psychiatric care, ensuring that all aspects of the patient’s condition are accurately identified and addressed.

Individualized interventions: Tailored treatment plans are essential for addressing the unique challenges faced by patients with psychiatric conditions in the context of in-ternal diseases. These plans should include personalized medication selection, psychotherapy adapted to the patient’s needs, lifestyle modifications, and supportive interventions designed to meet the multifaceted requirements of the individual.

Collaborative care: Effective management hinges on interprofessional collaboration and communication among healthcare providers. Primary care physicians, specialists, psychiatrists, psychologists, social workers, and allied health professionals must work together to deliver coordinated and holistic care that addresses the diverse and interconnected needs of these patients.

Diagnosing and managing psychiatric conditions in patients with internal diseases demands a nuanced understanding of the intricate relationship between physical and mental health. By employing multidisciplinary approaches that prioritize individualized care, healthcare professionals can effectively address the challenges associated with these comorbidities. Such strategies not only improve the quality of care but also enhance patient outcomes and overall well-being.

### 2.11. Diagnostic and Treatment Considerations

Diagnosing psychiatric conditions in patients with internal diseases requires a nuanced approach that accounts for the complexities of symptom presentation, overlapping manifestations of physical and mental health, and the influence of the underlying medical condition on mental status. A thorough and integrated diagnostic process is essential to address these challenges effectively.

Differential diagnosis: Accurate differentiation between primary psychiatric conditions and secondary manifestations related to the underlying medical condition is critical. Comprehensive assessments should evaluate the nature of the patient’s symptoms while accounting for potential overlaps between psychiatric and physical health domains.

Atypical presentations: Psychiatric conditions in patients with internal diseases often deviate from traditional symptom profiles, requiring heightened clinical vigilance. For example, depression in patients with chronic pain may manifest as irritability or somatic complaints rather than classic mood disturbances, complicating timely recognition and diagnosis.

Medical comorbidities: The presence of medical comorbidities can obscure the clinical picture, as certain internal diseases produce neuropsychiatric symptoms that may mimic primary psychiatric disorders. Clinicians must evaluate the impact of the underlying medical condition on the patient’s mental health to ensure a comprehensive diagnostic approach.

Specialized assessment tools: Complex presentations may necessitate the use of specialized diagnostic tools, such as cognitive screening instruments and structured interviews. These tools provide valuable insights into the nature and severity of symptoms, aiding in the distinction between primary psychiatric disorders and secondary manifestations.

Diagnostic collaboration: Interdisciplinary collaboration between medical and psychiatric specialists is essential for comprehensive evaluation and accurate diagnosis. Drawing on the expertise of both disciplines ensures a nuanced understanding of the interplay between physical and mental health, facilitating informed and holistic care planning.

Effective diagnosis and treatment of psychiatric conditions in patients with internal diseases requires careful consideration of the interplay between physical and mental health. By leveraging differential diagnosis, recognizing atypical presentations, and fostering interdisciplinary collaboration, clinicians can improve diagnostic accuracy and optimize patient outcomes.

### 2.12. Treatment Considerations

Effectively managing psychiatric conditions in patients with internal diseases re-quires a nuanced approach that addresses the complexities of pharmacological interactions, disease-specific symptoms, and adherence barriers. Key considerations include medication safety, tailored interventions, and personalized care plans.

Medication interactions: Psychiatric medications must be carefully evaluated for potential interactions with treatments for the primary medical condition [48]. Clinicians should consider the pharmacological implications of psychotropic medications in the context of the patient’s medical comorbidities, aiming to minimize adverse drug interactions and treatment-related complications.

Disease-specific symptoms: Tailoring treatment to address neuropsychiatric symptoms associated with specific medical conditions is crucial [2]. Clinical strategies should align with the distinct features of psychiatric manifestations linked to the underlying disease, requiring targeted interventions that address the unique characteristics of each patient’s presentation.

Individualized treatment plans: Personalizing treatment plans is essential to accommodate the intricate relationship between physical and mental health. Multimodal interventions, including pharmacological therapies, psychotherapeutic approaches, and supportive measures, should be tailored to the patient’s unique circumstances, ensuring comprehensive and effective care.

Treatment adherence: Recognizing and addressing barriers to treatment adherence is critical for optimizing outcomes. Cognitive impairments, physical limitations, and psychological factors related to chronic illness can hinder adherence to treatment regimens [49]. Integrating strategies that address these barriers into the treatment plan is vital for ensuring sustained therapeutic engagement.

Diagnosing and treating psychiatric conditions in patients with internal diseases requires a deep understanding of the interplay between physical and mental health. These considerations highlight the need for a comprehensive, patient-centered approach involving collaboration among healthcare providers, tailored interventions, and meticulous attention to the complexities of managing psychiatric conditions in the context of medical comorbidities. By addressing these challenges, clinicians can enhance outcomes and im-prove the overall quality of care for this population.

### 2.13. Treatment Approaches for Psychiatric Disorders in the Context of Internal Medicine: Challenges and Considerations

Managing psychiatric conditions in patients with internal diseases requires a nuanced, multidisciplinary approach that balances the complexities of mental healthcare with the challenges posed by medical comorbidities. Effective treatment strategies must address medication interactions, disease-specific symptoms, and the broader psychosocial context. The following approaches outline key considerations for optimizing care in this population.

#### 2.13.1. Pharmacotherapy

- Selection of psychotropic medications: Clinicians must carefully evaluate potential interactions between psychotropic drugs and treatments for internal diseases. For in-stance, tricyclic antidepressants may increase the risk of cardiovascular complications in patients with cardiac comorbidities, whereas selective serotonin reuptake inhibitors (SSRIs) or serotonin–norepinephrine reuptake inhibitors (SNRIs) are generally safer alter-natives [50,51].
- Dose adjustments: Physiological changes or altered drug metabolism associated with internal diseases may necessitate dose adjustments or specialized monitoring of psychotropic medications [52,53]. Collaborative decision-making between psychiatric and medical specialists is essential to establish safe and effective regimens.
- Adherence and monitoring: Regular assessment of medication adherence and therapeutic response is critical in patients with complex comorbidities. Close communication between the patient, psychiatric team, and medical providers ensures early identification of adverse effects or drug interactions.

#### 2.13.2. Psychotherapy

- Cognitive Behavioral Therapy (CBT) and adaptations: CBT is effective in addressing psychiatric symptoms in patients with internal diseases, particularly when tailored to accommodate physical limitations, cognitive impairments, or disease-related stressors [54,55]. Adapting psychotherapeutic techniques to align with the patient’s medical condition enhances their applicability and impact.
- Collaborative care: Integrating psychotherapeutic interventions within collaborative care models ensures a comprehensive understanding of the patient’s physical and mental health needs [56]. This approach promotes coordinated and individualized treatment strategies.

#### 2.13.3. Lifestyle Interventions

- Exercise and physical activity: Structured exercise programs tailored to the patient’s medical condition and physical capabilities can improve both mental and physical health outcomes [57]. Collaboration between psychiatric and medical teams is essential for implementing safe and effective regimens.
- Nutritional interventions: Addressing nutritional deficiencies and promoting dietary modifications tailored to the patient’s medical condition can support holistic treatment approaches and improve overall well-being [58].

#### 2.13.4. Supportive Interventions

- Support groups: Participation in support groups tailored to individuals with specific medical and psychiatric comorbidities provides emotional support, education, and a sense of community [59,60]. These groups can be facilitated in collaboration with psychiatric and medical care providers.
- Caregiver support: Supporting caregivers and family members is vital in managing the complex needs of patients. Providing resources and guidance can help mitigate caregiver stress and enhance overall patient care.

#### 2.13.5. Integrative Medicine

- Mind–body interventions: Techniques such as mindfulness meditation, yoga, and relaxation strategies can complement psychiatric care by addressing stress and enhancing mental health outcomes [61]. Collaborative discussions between psychiatric and medical providers are necessary to ensure safe implementation.
- Herbal and dietary supplements: The use of herbal remedies and supplements should be carefully evaluated for potential interactions with medical treatments [62]. Collaborative decision-making ensures safety and efficacy in this context.

Treating psychiatric conditions in patients with internal diseases requires a holistic, patient-centered approach that integrates pharmacological, psychotherapeutic, lifestyle, and supportive interventions. Collaboration among psychiatric and medical specialists is essential for developing tailored treatment plans that address the complexities of comorbid physical and mental health conditions. By prioritizing individualized care and interdisciplinary teamwork, clinicians can optimize outcomes and improve the overall well-being of this population.

## 3. Discussion

Psychiatric conditions significantly influence the prognosis and outcomes of patients with internal diseases, creating a complex bidirectional interplay that exacerbates morbidity, reduces treatment adherence, and diminishes quality of life [63]. This relationship is underpinned by shared physiological, neuroinflammatory, and psychosocial mechanisms, which highlight the need for actionable, evidence-based interventions in clinical practice.

### 3.1. Mechanistic Pathways Underlying Comorbidities

The interplay between psychiatric and internal medical conditions is driven by intricate physiological and neuroinflammatory mechanisms [64]. Chronic inflammatory states, such as those observed in rheumatoid arthritis, inflammatory bowel disease, and systemic lupus erythematosus, are strongly associated with elevated levels of pro-inflammatory cytokines, including IL-6 and TNF-alpha [65]. These cytokines disrupt neurotransmitter pathways, contributing to the onset and persistence of psychiatric conditions like depression and anxiety.

Stress-related dysregulation of the hypothalamic–pituitary–adrenal (HPA) axis further compounds these effects, leading to metabolic disturbances often seen in conditions such as diabetes and cardiovascular diseases [66,67,68,69]. For instance, individuals with diabetes and coexisting depression frequently exhibit worse glycemic control, which is mediated by chronic stress and its downstream effects on insulin sensitivity.

Emerging evidence also highlights the role of gut–brain interactions in this bidirectional relationship [70,71]. Alterations in gut microbiota composition are associated with increased systemic inflammation and psychiatric symptoms [72,73,74,75]. Interventions targeting the microbiome, such as dietary modifications or probiotics, may offer promising avenues for integrated care, addressing both physical and mental health outcomes.

### 3.2. Diagnostic Challenges in Complex Comorbidities

The overlapping symptoms of psychiatric and internal medical conditions pose significant diagnostic challenges, often leading to delayed or inaccurate diagnoses [76]. Common symptoms such as fatigue, cognitive impairments, and sleep disturbances may be misattributed to the primary medical condition, masking underlying psychiatric dis-orders. This diagnostic ambiguity is particularly evident in chronic illnesses like hypothyroidism and chronic kidney disease, where depressive or anxious states are frequently overlooked [77,78].

Integrated diagnostic tools, combining physical and mental health assessments, are essential for overcoming these challenges. The adaptation of screening tools like the PHQ-9 and GAD-7 in the context of chronic diseases is a critical step in ensuring early detection and intervention [79]. Additionally, interdisciplinary collaborations between psychiatrists and internists are vital for nuanced diagnostic evaluations, particularly in distinguishing primary psychiatric conditions from secondary neuropsychiatric manifestations, such as hepatic encephalopathy or steroid-induced psychosis [80,81].

### 3.3. Psychosocial Mechanisms and Patient Outcomes

Beyond physiological pathways, psychosocial factors play a pivotal role in shaping the outcomes of patients with comorbid psychiatric and internal medical conditions [82]. Stigma surrounding mental health remains a significant barrier to care, particularly in populations where psychological distress is culturally stigmatized [83,84,85]. This can lead to delayed treatment-seeking behaviors and poorer adherence to therapeutic regimens, exacerbating both psychiatric and physical health outcomes.

Socioeconomic determinants further amplify these challenges. Patients with limited access to healthcare, financial instability, or low educational attainment are disproportionately affected, often experiencing a cycle of worsening mental and physical health [86]. Addressing these psychosocial determinants through community-based interventions, enhanced access to care, and patient education programs is crucial for improving outcomes.

### 3.4. Addressing Research Gaps in Mental Healthcare for Patients with Internal Diseases

While significant progress has been made in understanding the intersection of mental health and internal diseases, several gaps in current research warrant further investigation. To address these gaps, specific study designs and frameworks are proposed to guide future research and enhance our understanding of this complex interplay.

Mechanistic understanding: A combination of translational research and experimental studies can be employed to investigate the underlying pathophysiological mechanisms. Animal models and cellular studies could explore the role of neuroinflammation, neuroendocrine dysregulation, and microbiome changes in mediating the bidirectional relationship between psychiatric conditions and internal diseases. Clinical studies using advanced imaging techniques, such as functional MRI or PET scans, could complement these findings by identifying the biomarkers of these interactions in patients.

Longitudinal studies: Future research should prioritize long-term, prospective cohort studies that track patients with comorbid psychiatric and internal diseases over time. These studies should include standardized measures for mental health symptoms and disease progression, as well as data on treatment adherence and healthcare utilization. For example, a 10-year cohort study could examine the reciprocal impact of depression on diabetes control and the effect of diabetes management on depressive symptoms.

Efficacy of integrated care models: Randomized controlled trials (RCTs) are essential to evaluate the effectiveness and cost-efficiency of integrated care models. These trials should compare different models, such as collaborative care versus co-located care, and assess their impact on patient outcomes, healthcare costs, and satisfaction. For example, an RCT could compare outcomes in patients receiving integrated psychiatric and medical care versus standard care.

Precision medicine approaches: Cross-sectional studies and multiomics approaches could help identify genetic, proteomic, and metabolomic biomarkers associated with specific psychiatric and internal disease comorbidities. A precision medicine framework could be developed by integrating patient data from electronic health records (EHRs) with genomic and lifestyle data, enabling personalized treatment plans.

Mental health screening tools: Mixed-methods research could be used to develop and validate disease-specific screening tools. Qualitative studies involving patients and clinicians could inform the design of these tools, while quantitative studies could assess their sensitivity, specificity, and usability in different clinical settings. For example, a screening tool tailored for cancer patients could assess anxiety and depression levels during different stages of treatment.

Health economics research: Decision-analytic modeling studies could assess the cost-effectiveness of interventions targeting psychiatric comorbidities in internal diseases. These models could incorporate direct and indirect costs, such as healthcare expenses and loss of productivity, to guide resource allocation. For instance, a cost-effectiveness analysis could compare collaborative care programs to standard care for patients with diabetes and depression.

Psychosocial determinants: Community-based participatory research (CBPR) frame-works could be used to explore the influence of psychosocial factors such as stigma, socioeconomic status, and social support. These studies could involve stakeholders, including patients and community organizations, to develop culturally sensitive interventions aimed at improving mental health outcomes.

Impact of treatment modalities: Comparative effectiveness research (CER) could evaluate how different medical treatments for internal diseases influence psychiatric symptoms, and vice versa. For example, a CER study could assess the impact of insulin therapy versus newer diabetes medications on depression outcomes, while also examining how antidepressants affect glycemic control.

By proposing these study designs and frameworks, we aim to guide future research efforts in addressing the identified gaps. These approaches can facilitate the development of effective interventions and personalized care strategies, ultimately improving the quality of life for individuals with comorbid physical and mental health challenges.

### 3.5. Bridging the Gap Between Primary and Tertiary Care

Establishing Coordination Mechanisms:

Implement standardized referral systems to ensure the efficient transfer of patient information and seamless transitions across care levels.

Develop multidisciplinary teams, including primary care physicians, specialists, psychiatrists, and social workers, to provide holistic care planning and management.

Training Programs for Primary Care Providers:

Equip primary care practitioners with the necessary skills to identify psychiatric comorbidities early using validated screening tools such as the PHQ-9 and GAD-7.

Offer continuous professional development programs tailored to address common challenges in managing comorbid physical and psychiatric conditions.

Liaison Psychiatry Services:

Integrate liaison psychiatry into primary care settings to provide immediate support for complex cases and to enhance the collaboration between mental health and medical care teams.

Infrastructure and Resource Allocation:

Invest in telemedicine and electronic health records (EHRs) to bridge geographical and logistical barriers, particularly in resource-limited settings.

Expand mental health services to underserved areas by allocating resources to primary care clinics for co-located psychiatric services.

Adapting Models to National Contexts:

In high-income countries, focus on co-located care models that integrate mental health professionals directly into primary care settings.

In low- and middle-income countries, prioritize mobile health initiatives and task-shifting strategies, training community health workers to provide basic mental healthcare and coordinate with tertiary centers when needed.

Monitoring and Quality Improvement:

Establish mechanisms to monitor patient outcomes, feedback loops, and shared metrics of success between primary and tertiary care systems.

Encourage research into the cost-effectiveness and clinical impact of integrated care models across diverse healthcare systems.

### 3.6. Proposing Potential Strategies and Recommendations for Healthcare Providers to Improve Holistic Care

To enhance the holistic care of patients with both medical and mental health needs, clinicians can implement several strategies aimed at operationalizing multidisciplinary approaches. Integrated care models are a critical component, incorporating mechanisms such as liaison services, shared care agreements, and co-located care teams. Liaison ser-vices involve establishing dedicated teams of psychiatrists, psychologists, and medical specialists to address cases requiring integration between mental health and medical care. These teams can function effectively within hospitals or outpatient clinics. Shared care agreements formalize collaboration between primary care providers and mental health professionals, ensuring continuity of care, regular communication, and shared responsibility for patient management. Similarly, co-located care teams, where mental health and medical professionals work in the same physical space, promote direct collaboration and reduce fragmentation in patient care.

Liaison psychiatry involves integrating mental health professionals into general medical settings to address the complex interplay between physical and mental health. The model typically includes consultation-liaison services, psychiatric support for medical staff, and the co-management of patients with comorbid medical and psychiatric conditions. Evidence-based benefits of liaison psychiatry include improved diagnosis and treatment of psychiatric disorders in medical settings, enhanced patient outcomes, reduced hospital readmissions, and potential cost savings [87].

However, the feasibility of implementing liaison psychiatry depends on several fac-tors, including resource availability, institutional support, and the specific needs of the healthcare system. Resource implications include the need for specialized training, dedicated funding, and ongoing support for interdisciplinary collaboration [88,89,90].

Despite these challenges, liaison psychiatry offers significant potential for improving the integration of mental and physical healthcare, fostering a holistic approach to patient management [89]. By addressing psychiatric conditions in medical settings, clinicians can enhance overall patient care and outcomes.

Screening and early intervention are essential components of holistic care. Structured screening protocols that incorporate tools like the PHQ-9 for depression and the GAD-7 for anxiety should be integrated into routine assessments within internal medicine clinics. Clear follow-up pathways must also be established, enabling direct referrals to mental health services for patients identified through these screenings.

A multidisciplinary team approach further enhances patient care by fostering collab-oration across disciplines. Regular case conferences provide a platform for physicians, psychiatrists, psychologists, social workers, and allied health professionals to discuss complex cases. Care coordinators play a crucial role in overseeing the integration of care plans, while the inclusion of patient and family representatives ensures that holistic perspectives are considered in decision-making processes.

Personalized treatment plans are vital for addressing the unique needs of patients. These plans should combine pharmacological interventions tailored to minimize drug–drug interactions, evidence-based psychotherapeutic approaches like cognitive-behavioral therapy (CBT), and lifestyle modifications, including exercise, nutrition, and stress management programs. These interventions must be adapted to accommodate patients’ physical limitations and cognitive impairments.

Patient education and empowerment are also essential for improving outcomes. Educational workshops and modules should focus on the bidirectional impact of physical and mental health, self-management strategies for medication adherence and stress reduction, and the provision of customized resources tailored to the patient’s condition.

To ensure healthcare providers are equipped to address psychiatric symptoms, mental health training for medical providers is imperative. Workshops and professional development courses should emphasize recognizing atypical presentations of psychiatric symptoms, employing sensitive communication techniques, and utilizing decision-support tools to manage psychiatric comorbidities effectively.

Telemedicine and remote monitoring represent innovative approaches to extending mental healthcare. Telehealth programs can integrate mental health services into follow-up care for internal diseases, while remote monitoring tools, such as mobile apps or wearable devices, enable the real-time tracking of symptoms and early intervention. Alerts generated by these tools can facilitate prompt responses by medical and mental health teams.

Supportive services integration is another key strategy. Collaborations with community organizations address social determinants of health, such as housing and employment, while case managers help patients navigate healthcare systems and access necessary mental health resources. Additionally, research and data sharing across institutions can improve care delivery. Collaborative networks allow for the analysis of integrated care outcomes, identification of patterns in comorbid conditions, and creation of shared registries to streamline research and care coordination.

Finally, care coordination and transition planning are crucial for ensuring seamless patient experiences. Detailed discharge plans that include psychiatric follow-up and dedicated transition coordinators facilitate smooth handoffs between hospital and outpatient settings, ensuring the continuity of care.

By implementing these strategies, healthcare providers can enhance the multidisciplinary care of patients with complex medical and mental health needs, ensuring comprehensive support and improved outcomes.

## 4. Conclusions

Our review highlights the critical intersection between psychiatric conditions and in-ternal diseases, emphasizing the importance of recognizing and addressing psychiatric comorbidities in this population. Key insights include the following.

Complex interplay: Psychiatric conditions and internal diseases share a bidirectional and complex relationship, each influencing the progression, management, and outcomes of the other. Understanding this interplay is fundamental to delivering comprehensive care.

Diagnostic challenges: Atypical or masked presentations of psychiatric symptoms in patients with internal diseases often lead to diagnostic delays. These challenges under-score the need for heightened clinical vigilance and the application of specialized assessment techniques.

Impact on prognosis and outcomes: Psychiatric conditions can significantly exacerbate the burden of internal diseases, affecting treatment adherence, disease progression and quality of life. Addressing these mental health needs is essential for improving prognosis and overall health outcomes.

Personalized treatment strategies: Individualized care plans are critical for addressing the unique challenges posed by comorbid medical and psychiatric conditions. These strategies should encompass optimized medication selection, psychotherapeutic approaches, lifestyle modifications, and supportive care tailored to the patient’s specific needs.

Multidisciplinary collaboration: Effective care for patients with comorbidities re-quires a multidisciplinary approach involving collaboration among medical, psychiatric, and allied health professionals. This integrated model ensures a holistic and patient-centered approach to managing interconnected physical and mental health concerns.

Longitudinal impact: Prospective, long-term studies are needed to better understand the trajectory of psychiatric symptoms in patients with internal diseases. These studies can inform clinical strategies and shed light on the long-term impact of comorbidities on disease progression and patient outcomes.

Precision medicine and integrated care: Research into precision medicine and integrated care models has the potential to revolutionize treatment approaches for patients with comorbidities. These advancements can optimize outcomes, minimize adverse effects, and set new standards for comprehensive care delivery.

In summary, the proactive recognition and management of psychiatric conditions in patients with internal diseases are vital for enhancing treatment outcomes and overall well-being. By addressing the intricate interplay between physical and mental health, and by adopting personalized, multidisciplinary, and integrated care strategies, healthcare professionals can significantly improve the quality of care for this vulnerable population. Prioritizing the integration of psychiatric and medical care is essential for achieving better health outcomes and fostering holistic patient well-being.

Despite the growing recognition of the interplay between psychiatric disorders and internal diseases, significant gaps remain in the literature regarding the integration of care across primary and tertiary levels. Current research often focuses on isolated aspects of these conditions—such as their pathophysiology or treatment modalities—without addressing the systemic challenges of care coordination.

Patients with comorbid psychiatric and medical conditions frequently experience fragmented care, leading to delayed diagnoses, suboptimal treatment outcomes, and increased healthcare utilization. For instance, primary care providers may lack the resources or specialized training to identify complex psychiatric symptoms masked by medical conditions. Conversely, tertiary care settings often overlook the broader psychosocial and primary care context, resulting in a disconnect that hinders holistic patient management.

## 5. Future Directions

The intricate relationship between psychiatric conditions and internal diseases necessitates ongoing research and innovation to enhance patient care and address existing knowledge gaps. This review identifies several key areas for future exploration.

### 5.1. Mechanistic Pathways of Interaction

Further investigation into the physiological, psychological, and environmental fac-tors underlying the bidirectional relationship between psychiatric and internal diseases is critical. Research on mechanisms such as neuroinflammation, neuroendocrine dysregulation, and psychosocial stress can inform the development of novel therapeutic approaches.

### 5.2. Enhancing Multidisciplinary and Integrated Care Models

Developing and evaluating integrated care models that promote collaboration among internal medicine specialists, psychiatrists, and allied health professionals is essential. These models should prioritize a holistic approach that addresses both physical and mental health needs, with a focus on improving clinical and psychosocial outcomes.

### 5.3. Advancing Diagnostic and Management Strategies

Future research should aim to create advanced diagnostic tools and disease-specific screening protocols to facilitate the early detection of psychiatric symptoms in patients with internal diseases. Additionally, innovative management strategies that account for the unique challenges of comorbid conditions are needed to optimize patient care.

### 5.4. Targeting Research Gaps with Longitudinal and Precision Medicine Approaches

Longitudinal studies are crucial for understanding the long-term impact of psychiatric conditions on the progression and prognosis of internal diseases. Precision medicine approaches that leverage biomarkers, genetic profiling, and individual patient characteristics can support the development of tailored interventions that address specific needs. For example, identifying patient subgroups based on genetic predisposition or specific biomarkers may allow for targeted pharmacological or psychotherapeutic treatments.

### 5.5. Leveraging Technology for Holistic Patient Care

The integration of telemedicine, digital health platforms can enhance accessibility, continuity of care, and treatment adherence. These technologies hold significant potential for improving patient outcomes and streamlining care delivery. Additionally, wearable devices and mobile health applications can provide real-time monitoring of mental and physical health parameters, facilitating early intervention and personalized care.

### 5.6. Exploring Emerging Areas: Precision Psychiatry and Digital Health Tools

Emerging research areas such as precision psychiatry and digital health tools are particularly promising for integrated care. Precision psychiatry aims to customize mental health interventions by incorporating insights from genomics, neuroimaging, and other advanced diagnostic tools, potentially transforming the management of comorbid conditions. Meanwhile, digital health tools—such as cognitive behavioral therapy (CBT)-based apps, virtual mental health platforms, and remote monitoring systems—can complement traditional care, providing scalable and accessible solutions for patients with limited access to in-person services.

### 5.7. Addressing Psychosocial and Environmental Factors

The influence of psychosocial determinants, such as stigma, social support, and socioeconomic factors, on mental health outcomes warrants further exploration. Targeted interventions that address these factors can significantly improve resilience and quality of life among affected patients.

### 5.8. Developing Actionable Strategies for Clinical Practice

Translating research findings into practical clinical strategies is imperative. Future efforts should equip clinicians with evidence-based tools, training, and recommendations to effectively manage the complexities of comorbid psychiatric and internal diseases.

By focusing on these research directions, future studies can bridge critical gaps in understanding and enhance holistic, patient-centered care. These efforts will foster the integration of mental and physical healthcare, optimize clinical and psychosocial out-comes, and contribute to an improved quality of life for patients living with comorbid psychiatric and internal diseases.

## Data Availability

We confirm that the main data supporting the findings of this study are available within the article, and any other additional data are available on request.
